# Socioeconomic Status, Self-Rated Health, and Mortality in a Multiethnic Sample of US Adults

**DOI:** 10.2188/jea.JE20100142

**Published:** 2011-09-05

**Authors:** Sivaranjani Suresh, Charumathi Sabanayagam, Anoop Shankar

**Affiliations:** 1 University Scholars Programme, National University of Singapore, Singapore; 2 Department of Community Medicine, West Virginia University School of Medicine, Morgantown, WV, USA

**Keywords:** education, income, self-rating of health, ethnicity, mortality, United States

## Abstract

**Objective:**

To examine the association between socioeconomic status (SES), self-rated health (SRH), and mortality separately by race-ethnicity in a nationally representative sample of US adults.

**Methods:**

We analyzed data from 16 716 adult women and men who were followed up for mortality for up to 12 years as part of the third National Health and Nutrition Examination survey (NHANES III). Poverty-income ratio (PIR) and education were assessed as measures of SES. All-cause mortality (*n* = 2850) was recorded from the NHANES III linked mortality file.

**Results:**

Lower PIR was associated with mortality after adjustment for lifestyle, clinical risk factors, and SRH in all racial-ethnic groups (*P*-trend <0.005). In contrast, after adjusting for lifestyle and clinical risk factors, lower education was not associated with all-cause mortality in non-Hispanic whites (*P*-trend = 0.16), whereas the association remained significant after adjustment for SRH and lifestyle and clinical risk factors in other race-ethnicities (*P*-trend = 0.005; P-interaction between education categories and race-ethnicity was 0.02).

**Conclusions:**

Our results suggest that lower PIR was associated with mortality in all racial-ethnic groups. In contrast, lower education was significantly associated with mortality only in racial-ethnic groups other than non-Hispanic whites. Our results indicate that, beyond lifestyle and clinical risk factors, adjusting for SRH resulted in only a modest change in the association of SES and mortality.

## INTRODUCTION

The effect of socioeconomic disparities on health outcomes is a public health concern. Major socioeconomic variables include income, education, occupation, and insurance status, all of which may dictate differential access and control of material and social resources in a society. Of particular interest is the manner in which other global inequalities, such as ethnic and sex inequalities, interact with socioeconomic inequalities to modify associations with health. Although ethnic and sex disparities in health have been observed and extensively studied,^1^^–^^5^ a side-by-side comparison of race-ethnicity, sex, and socioeconomic disparities would likely better illuminate the particular effects on specific segments of a population, which is crucial for targeted public health decision-making.

An understanding of the pathways and mechanisms through which health is affected is of value to public health. To date, research suggests that individual health behaviors (eg, smoking) and clinically diagnosed health status (eg, high blood pressure)^6^ are variables that might partially explain the observed social gradient in health, as these variables also demonstrate a similar socioeconomic gradient.^7^^–^^9^ However, it is possible that subjective factors such as psychosocial and emotional components of health are also determinants of the social gradient in health.^10^^,^^11^

Global self-rating of health has been widely advanced as a reliable predictor of mortality in several studies.^12^^,^^13^ As opposed to conventional objective assessments, self-rated health (SRH) considers a more holistic approach to health assessment by accounting for physical, social, and emotional influences on health.^12^^–^^15^ Socioeconomic differentials have been established in SRH^16^^–^^18^: self-rated health in lower socioeconomic groups is generally worse than that in higher socioeconomic groups, and several studies have also demonstrated SRH variations across sex and ethnicities.^12^^,^^13^ However, little research has evaluated the role of SRH in explaining socioeconomic differentials in mortality. A study conducted in the EPIC-Norfolk cohort concluded that SRH does not explain socioeconomic differentials in mortality beyond those already explained by clinical and lifestyle-related factors in the European cohort.^19^ However, that study did not conduct any subgroup analyses (eg, by race-ethnicity) and examined only occupation status as a socioeconomic variable. It has been suggested that income may be a better socioeconomic status (SES) variable of health than occupation status.^20^ Furthermore, no studies have examined this relation in US cohorts. In this context, we examined the association between SES and all-cause mortality after adjustment for SRH, in addition to behavioral and clinical factors, in a large, nationally representative sample of US adults. We also performed stratified analyses by race-ethnicity to examine if there are racial differences in the observed associations.

## METHODS

### Study participants

The third National Health and Nutrition Examination survey (NHANES III) collected data on a nationwide probability sample of the civilian noninstitutionalized US population. Standardized questionnaires were administered at home, followed by detailed physical examinations at a mobile examination center. Detailed descriptions of the complex survey design, interviewing procedures, and physical examinations conducted have been published before and are available online.^21^ In brief, NHANES III uses a stratified multistage probability sample of households, with oversampling of Mexican Americans and non-Hispanic blacks to ensure adequate sample size for analysis. The study protocol was approved by the institutional review board of the National Center for Health Statistics of the US Centers for Disease Control and Prevention.

Of the 18 825 participants aged 20 years or older who participated in the interview and examination components of NHANES III, mortality assessment data were available for 18 800 participants. We excluded those with missing data on poverty-income ratio (PIR, *n* = 1978), education status (*n* = 96), and certain other variables included in the multivariable model (*n* = 10), leaving 16 716 for the current analysis.

### Outcome of interest

The main outcome of interest was all-cause mortality. Mortality was recorded from the NHANES III linked mortality file provided by the National Center for Health Statistics (NCHS).^22^ Mortality assessments were conducted from the baseline interview between 1988–94 through 31 December 2006. Mortality ascertainment was based on a probabilistic match between NHANES III and National Death Index death certificate records.

### Measurement of exposure

PIR and educational status were chosen as measures of SES. PIR was computed as the ratio of the midpoint of the observed family income category to the family’s appropriate poverty threshold set by the US Census Bureau in a given calendar year. PIR is one of the best available indicators of SES and is widely used as an indicator of SES in many studies using the NHANES data.^23^^,^^24^ PIR is a more sensitive SES measure than income because it allows income data to be compared across NHANES survey years as the income thresholds are adjusted for inflation. A PIR of 1 indicates the official federal poverty threshold. According to the 2009 Federal Poverty Guidelines, this corresponds to an individual earning $10 830 per year, a couple earning $14 570 per year, and a family of 3 earning $18 310 per year.^25^ Accordingly, a PIR lower than 1 is defined as poor, 1 to 1.9 as near poor, 2 to 3.9 as middle income, and 4 or higher as higher income. Educational status based on completed years of education was ascertained from the following questions, “What is the highest grade/year of regular school you have ever attended?” and “Did you finish that grade/year?”, with the response categorized as less than high school graduate (<12 years), high school graduate (12 years), and more than high school graduate (>12 years, including college degree). We also assessed insurance status, a strong correlate of income,^26^ as an alternative SES indicator. Participants were considered to have insurance coverage if they answered affirmatively to the question, “During the last month were you covered by one or more health insurance plans obtained privately or through an employer or union?”

### Measurement of covariates

Age, sex, race/ethnicity, smoking status, alcohol intake, self-reported history of diabetes, hypertension, coronary heart disease, stroke, cancer (excluding skin cancer), and intake of oral hypoglycemic drug or insulin administration or antihypertensive medication were assessed using a standardized questionnaire at home interview. Individuals who had smoked fewer than 100 cigarettes during their lifetime were considered never smokers, those who had smoked 100 or more cigarettes during their lifetime but did not currently smoke were considered former smokers, and those who had smoked 100 or more cigarettes during their lifetime and currently smoked were considered current smokers. Current alcohol drinking was defined as consumption of 1 or more alcoholic drink in the past month. One alcoholic drink was described as 360 mL of beer, 120 mL of wine, or 30 mL of hard liquor. SRH was assessed with the question, “Would you say your health in general is excellent, very good, good, fair or poor?” with the responses collapsed into 3 categories: excellent/very good, good, fair/poor. Information on anthropometric, physical, and laboratory measurements was obtained during the mobile examination center (MEC) examination.^27^ Blood pressure (BP) was measured using a mercury sphygmomanometer, and the average of 3 measurements was used as the systolic and diastolic BP values. Patients were considered to have hypertension if they reported taking antihypertensive medication, had a systolic BP of 140 mm Hg or higher, or a diastolic BP of 90 mm Hg or higher. Body mass index (BMI) was calculated as weight in kilograms divided by the square of the height in meters. Diabetes was defined as a serum glucose level of 126 mg/dL or higher after fasting for a minimum of 8 hours, a serum glucose level of 200 mg/dL or higher after fasting less than 8 hours before their NHANES visit, self-reported physician-diagnosed diabetes, or current use of oral hypoglycemic medication or insulin. Serum total and high-density lipoprotein (HDL) cholesterol levels were measured enzymatically. Cardiovascular disease (CVD) was defined as self-reported history of coronary heart disease, myocardial infarction, angina, or stroke. Participants were considered physically active if they walked a mile or more without stopping at least 20 times in the past month.

### Statistical analysis

In the current analysis, PIR values were categorized into quartiles. We compared selected characteristics of the participants by quartiles of PIR using the chi-square test or analysis of variance, as appropriate. We examined the association between PIR and all-cause mortality using logistic regression models. We calculated the odds ratio (OR) and 95% confidence interval (CI) of all-cause mortality associated with PIR quartiles using the highest quartile (quartile 4) as the referent in 3 nested models: (1) age- (years) and sex-adjusted model, (2) multivariable-adjusted model 1 additionally adjusted for race-ethnicity (non-Hispanic whites, non-Hispanic blacks, Mexican Americans, others), smoking (never, former, current), alcohol intake (absent, present), hypertension (absent, present), diabetes (absent, present), cancer (absent, present), CVD (absent, present), mean arterial BP (mm Hg), BMI (kg/m^2^), HDL cholesterol (mg/dL), and physical activity (absent, present), and (3) multivariable-adjusted model 2 adjusted for all variables in multivariable model 1 plus SRH (excellent/very good, good, fair/poor). We then examined the association between education and all-cause mortality using “more than high school graduate” as the reference category, using similar multivariable models. In subgroup analyses, we examined the association between the 2 SES indicators (PIR and education) and all-cause mortality stratified by sex and race-ethnicity. To examine the dose-response association between SES and all-cause mortality, we carried out statistical tests for linear trend by modeling each SES indicator category as an ordinal variable separately in the corresponding multivariable model. Interactions between SES measures (PIR and education) and sex and race-ethnicity were formally evaluated by including cross-product interaction terms in the corresponding multivariable models. In a supplementary analysis, we examined the association between SES and all-cause mortality using insurance status as an alternative SES indicator in the same multivariable models. Sample weights that accounted for the unequal probabilities of selection, oversampling, and nonresponse were applied to all analyses, using SUDAAN (version 8.0; Research Triangle Institute, Research Triangle Park, NC, USA) and SAS (version 9.2; SAS Institute, Cary, NC, USA) software; standard errors (SEs) were estimated using the Taylor series linearization method.

## RESULTS

Table 1 shows the characteristics of the study population by quartiles of PIR. Adults in the lowest PIR quartile: were more likely to be younger, female, less than high school-educated, and current smokers; had higher BMI and HDL cholesterol; had higher prevalences of diabetes, hypertension, and poor SRH; and were less likely to be non-Hispanic whites, physically active, and have insurance coverage. Table 2 shows the characteristics of the study population by educational categories. Adults with less than a high school education: were more likely to be older and current smokers; had higher BMI, systolic BP, and HDL cholesterol; had higher prevalences of diabetes, hypertension, CVD, cancer, and poor SRH; and were less likely to be non-Hispanic whites, current drinkers, physically active, and have insurance coverage.

**Table 1.** Baseline characteristics of the study population by quartiles of poverty-income ratio

	Poverty-income ratio quartiles				*P*-value
	
	Quartile 4 (3.4–11.9)	Quartile 3 (2.1–3.3)	Quartile 2 (1.2–2.0)	Quartile 1 (0–1.1)	
*n*	4180	4238	4111	4187	
Age, y	44.7 ± 0.5	43.7 ± 0.4	45.6 ± 0.8	42.7 ± 0.7	<0.0001
Females, %	49.6 ± 0.8	50.0 ± 0.9	55.0 ± 1.0	59.5 ± 1.3	<0.0001
Race-ethnicity, %					
Non-Hispanic whites	89.7 ± 0.7	79.4 ± 1.6	68.5 ± 1.9	51.2 ± 3.1	<0.0001
Non-Hispanic blacks	5.3 ± 0.5	9.0 ± 0.7	14.3 ± 0.9	24.1 ± 1.7	
Mexican Americans	1.8 ± 0.2	3.4 ± 0.3	7.5 ± 0.7	13.0 ± 1.4	
Others	3.2 ± 0.6	8.3 ± 1.3	9.8 ± 1.8	11.7 ± 2.8	
Poverty-income ratio	5.1 ± 0.07	2.6 ± 0.01	1.5 ± 0.01	0.7 ± 0.01	<0.0001
Education, %					
<high school	7.8 ± 0.5	22.8 ± 1.2	40.0 ± 1.5	51.6 ± 1.6	<0.0001
High school	29.7 ± 1.1	39.6 ± 1.4	36.5 ± 1.4	30.9 ± 1.3	
>high school	62.6 ± 1.2	37.6 ± 1.5	23.4 ± 1.5	17.5 ± 1.5	
Any insurance, %	94.2 ± 0.7	84.7 ± 1.4	60.3 ± 1.8	32.1 ± 1.8	<0.0001
Current smoking, %	23.0 ± 1.1	28.3 ± 1.2	35.2 ± 1.3	37.5 ± 1.2	<0.0001
Current drinking, %	58.0 ± 1.4	47.0 ± 1.6	36.9 ± 2.3	40.4 ± 1.8	<0.0001
Physically active, %	12.4 ± 0.7	12.97 ± 0.8	13.5 ± 1.1	14.3 ± 1.1	0.4
Hypertension, %	29.0 ± 1.0	31.6 ± 1.1	36.6 ± 2.3	37.3 ± 1.2	<0.0001
Diabetes mellitus, %	6.3 ± 0.5	7.3 ± 0.7	9.1 ± 0.6	11.5 ± 0.8	<0.0001
Cardiovascular disease, %	3.4 ± 0.3	5.1 ± 0.4	9.3 ± 0.7	8.8 ± 0.8	<0.0001
Cancer, %	3.9 ± 0.3	3.7 ± 0.4	3.8 ± 0.5	4.1 ± 0.6	0.92
Poor self-rated health, %	7.5 ± 0.5	13.5 ± 1.0	22.2 ± 1.2	33.0 ± 1.5	<0.0001
Body mass index, kg/m^2^	26.1 ± 0.1	26.8 ± 0.2	26.7 ± 0.2	27.0 ± 0.2	<0.0001
Systolic blood Pressure, mm Hg	129.8 ± 1.7	134.2 ± 3.3	134.2 ± 3.3	130.3 ± 1.9	0.13
Diastolic blood Pressure, mm Hg	83.4 ± 1.7	87.2 ± 2.8	84.7 ± 3.5	82.1 ± 1.8	0.02
High-density lipoprotein cholesterol, mg/dL	89.3 ± 4.1	89.4 ± 6.4	95.2 ± 7.1	102.8 ± 7.3	0.01

Numbers in the table are means (standard error) for continuous variables or percentages for categorical variables.

*P*-values represent differences in means (SD) or proportions on analysis of variance or the chi-square test.

**Table 2.** Baseline characteristics of the study population by educational categories

	Less than high school	High school	More than high school	*P*-value
*n*	6706	5140	4870	
Age, y	50.6 ± 0.6	43.4 ± 0.5	41.4 ± 0.5	<0.0001
Females, %	50.4 ± 1.2	56.4 ± 0.8	49.5 ± 0.8	<0.0001
Race-ethnicity, %				<0.0001
Non-Hispanic whites	64.0 ± 2.6	79.7 ± 1.2	83.4 ± 1.0	
Non-Hispanic blacks	13.8 ± 0.9	11.8 ± 0.8	7.8 ± 0.6	
Mexican Americans	11.4 ± 1.1	3.5 ± 0.3	2.2 ± 0.2	
Others	10.8 ± 2.3	5.0 ± 0.6	6.6 ± 0.7	
Poverty-income ratio	1.9 ± 0.04	2.9 ± 0.05	4.0 ± 0.08	<0.0001
Any insurance, %	54.2 ± 2.1	77.8 ± 1.2	88.6 ± 0.5	<0.0001
Current smoking, %	36.9 ± 1.1	34.2 ± 1.2	19.8 ± 1.1	<0.0001
Current drinking, %	35.5 ± 1.6	46.1 ± 1.6	57.9 ± 1.4	<0.0001
Physically active, %	13.9 ± 0.9	12.0 ± 0.6	13.0 ± 0.8	0.1
Hypertension, %	42.5 ± 1.2	33.3 ± 1.3	25.6 ± 1.1	<0.0001
Diabetes mellitus, %	11.8 ± 0.7	7.6 ± 0.6	5.7 ± 0.5	<0.0001
Cardiovascular disease, %	11.4 ± 0.7	4.6 ± 0.4	3.4 ± 0.3	<0.0001
Cancer, %	5.1 ± 0.4	4.0 ± 0.4	3.0 ± 0.3	<0.0001
Poor self-rated health, %	32.7 ± 1.4	13.6 ± 0.7	7.1 ± 0.5	<0.0001
Body mass index, kg/m^2^	27.1 ± 0.1	27.0 ± 0.2	25.6 ± 0.1	<0.0001
Systolic blood Pressure, mm Hg	137.3 ± 2.1	133.9 ± 2.1	127.3 ± 1.8	<0.0001
Diastolic blood Pressure, mm Hg	84.8 ± 2.3	86.7 ± 2.2	82.7 ± 1.9	0.1
High-density lipoprotein cholesterol, mg/dL	96.9 ± 4.8	90.9 ± 5.1	90.9 ± 6.0	0.3

Numbers in the table are means (standard error) for continuous variables or percentages for categorical variables.

*P*-values represent differences in means or proportions on analysis of variance or the chi-square test.

The association between PIR and all-cause mortality is shown in Table 3. The crude mortality rate was highest among those in the lowest PIR quartile and lowest among those in the highest PIR quartile. In logistic regression models, lower PIR quartiles were associated with mortality in both the age- and sex-adjusted model and multivariable model 1 (which additionally adjusted for lifestyle and clinical risk factors). Although additional adjustment for SRH in multivariable model 2 attenuated this association, it remained significant. When stratified by race-ethnicity, the association between PIR quartiles and mortality was consistently present in all categories of race-ethnicity (P-interaction between PIR quartiles × race-ethnicity = 0.3 in multivariable model 2). The association between education and all-cause mortality is shown in Table 4. Similar to PIR, the crude mortality rate was highest among those with less than a high school education and lowest among those with more than a high school education. In logistic regression models, decreasing categories of education were associated with mortality in both the age-and sex-adjusted model and multivariate model 1. However, in contrast to the findings for PIR, additional adjustment for SRH in multivariable model 2 considerably attenuated the OR for low education, and the association became nonsignificant. When stratified by race-ethnicity, education was not associated with mortality among non-Hispanic whites after adjustment for lifestyle and clinical risk factors, whereas the association remained significant after adjustment for lifestyle and clinical risk factors and SRH in other race-ethnicities (P-interaction between education categories × race-ethnicity = 0.02 in multivariable model 2).

**Table 3.** Association between poverty-income ratio and mortality by race-ethnicity

Poverty-income ratio quartiles	Number of subjects	Mortality rate, %	Age-, sex-adjusted OR (95% CI)	Multivariable model 1^a^ OR (95% CI)	Multivariable model 2^b^ OR (95% CI)
Whole population (*n* = 16 716)					
Quartile 4 (3.4–11.9)	4180	6.7	1.00 (referent)	1.00 (referent)	1.00 (referent)
Quartile 3 (2.1–3.3)	4238	8.3	1.18 (0.97–1.43)	1.05 (0.85–1.29)	0.98 (0.79–1.23)
Quartile 2 (1.2–2.0)	4111	15.3	1.98 (1.61–2.44)	1.74 (1.39–2.17)	1.56 (1.25–1.95)
Quartile 1 (0–1.1)	4187	15.5	2.95 (2.22–3.94)	2.16 (1.59–2.94)	1.81 (1.32–2.48)
*P*-trend			<0.0001	<0.0001	<0.0001
Non-Hispanic whites (*n* = 7365)					
Quartile 4 (3.4–11.9)	2802	6.8	1.00 (referent)	1.00 (referent)	1.00 (referent)
Quartile 3 (2.1–3.3)	2196	9.1	1.18 (0.96–1.44)	1.00 (0.79–1.26)	0.93 (0.72–1.20)
Quartile 2 (1.2–2.0)	1526	18.1	1.99 (1.57–2.52)	1.68 (1.30–2.18)	1.50 (1.16–1.95)
Quartile 1 (0–1.1)	841	20.4	3.40 (2.38–4.85)	2.33 (1.56–3.48)	1.88 (1.25–2.84)
*P*-trend			<0.0001	<0.0001	0.0008
Other race-ethnicities (*n* = 9351)					
Quartile 4 (3.4–11.9)	1378	5.3	1.00 (referent)	1.00 (referent)	1.00 (referent)
Quartile 3 (2.1–3.3)	2042	5.2	1.05 (0.59–1.87)	1.24 (0.76–2.02)	1.20 (0.73–1.96)
Quartile 2 (1.2–2.0)	2585	9.2	1.71 (1.06–2.75)	1.87 (1.30–2.68)	1.72 (1.20–2.48)
Quartile 1 (0–1.1)	3346	10.3	2.16 (1.28–3.65)	2.13 (1.40–3.26)	1.92 (1.26–2.93)
*P*-trend			0.0001	0.0002	0.001

Abbreviations: CI, confidence interval; OR, odds ratio.

^a^Adjusted for age (years), sex (women, men), race-ethnicity (non-Hispanic whites, non-Hispanic blacks, Mexican Americans, others), smoking status (never, former, current), current drinker (absent, present), hypertension (absent, present), diabetes (absent, present), cancer (absent, present), cardiovascular disease (absent, present), mean arterial blood pressure (mm Hg), high-density lipoprotein cholesterol (mg/dL), body mass index (kg/m^2^), and physical activity (absent, present).

^b^Adjusted for all variables in multivariable model 1 plus self-rated health (excellent/very good, good, fair/poor).

P-interaction (poverty-income ratio quartiles × race-ethnicity) was 0.3 in multivariable model 2.

**Table 4.** Association between education and mortality by race-ethnicity

Education categories	Number of subjects	Mortality rate, %	Age-, sex-adjusted OR (95% CI)	Multivariable model 1^a^ OR (95% CI)	Multivariable model 2^b^ OR (95% CI)
Whole population (*n* = 16 716)					
>high school	4870	5.7	1.00 (referent)	1.00 (referent)	1.00 (referent)
high school	5140	8.9	1.40 (1.16–1.70)	1.21 (0.99–1.47)	1.12 (0.93–1.36)
<high school	6706	18.7	1.74 (1.45–2.07)	1.28 (1.06–1.54)	1.10 (0.91–1.32)
*P*-trend			<0.0001	0.009	0.33
Non-Hispanic whites (*n* = 7365)					
>high school	2746	6.2	1.00 (referent)	1.00 (referent)	1.00 (referent)
high school	2441	9.7	1.39 (1.11–1.73)	1.16 (0.91–1.47)	1.07 (0.84–1.36)
<high school	2178	22.1	1.63 (1.34–1.98)	1.17 (0.94–1.47)	0.99 (0.80–1.24)
*P*-trend			<0.0001	0.16	0.91
Other race-ethnicities (*n* = 9351)					
>high school	2124	3.7	1.00 (referent)	1.00 (referent)	1.00 (referent)
high school	2699	5.7	1.44 (0.92–2.24)	1.50 (1.03–2.19)	1.45 (0.99–2.10)
<high school	4528	12.8	1.99 (1.26–3.13)	1.98 (1.32–2.96)	1.80 (1.19–2.71)
*P*-trend			0.001	0.0007	0.005

Abbreviations: CI, confidence interval; OR, odds ratio.

^a^Adjusted for age (years), sex (women, men), race-ethnicity (non-Hispanic whites, non-Hispanic blacks, Mexican Americans, others), smoking status (never, former, current), current drinker (absent, present), hypertension (absent, present), diabetes (absent, present), cancer (absent, present), cardiovascular disease (absent, present), mean arterial blood pressure (mm Hg), high-density lipoprotein cholesterol (mg/dL), body mass index (kg/m^2^), and physical activity (absent, present).

^b^Adjusted for all variables in multivariable model 1 plus self-rated health (excellent/very good, good, fair/poor).

P-interaction (education categories × race-ethnicity) was 0.02 in multivariable model 2.

The association between SES indicators (PIR and education) and all-cause mortality stratified by sex and race-ethnicity is shown in Table 5. In men, the association between PIR and mortality was significant among both non-Hispanic whites and other race-ethnicities, whereas the association in women was not significant in either non-Hispanic whites or other race-ethnicities (P-interaction between PIR quartiles × sex = 0.1). Consistent with the main findings in Table 4, there was no significant association between education and mortality in either non-Hispanic white men or women, whereas a significant association was observed between education and mortality in both men and women of other race-ethnicities (P-interaction between education categories × sex = 0.8).

**Table 5.** Association between socioeconomic status and mortality stratified by sex and race-ethnicity

SES indicator	Non-Hispanic whites (*n* = 7365)			Other race-ethnicities (*n* = 9351)		
	
	Number of subjects	Mortality rate, %	Multivariable model^a^ OR (95% CI)	Number of subjects	Mortality rate, %	Multivariable model^a^ OR (95% CI)
Poverty-income ratio						
Men (*n* = 7894)						
Quartile 4	1382	7.7	1.00 (referent)	740	7.1	1.00 (referent)
Quartile 3	1070	10.1	1.03 (0.74–1.44)	1040	5.6	1.08 (0.66–1.75)
Quartile 2	664	18.9	1.70 (1.16–2.49)	1261	10.9	1.74 (1.16–2.60)
Quartile 1	309	19.8	2.27 (1.26–4.09)	1428	12.6	2.16 (1.26–3.69)
*P*-trend	3425		0.002	4469		0.002
Women (*n* = 8822)						
Quartile 4	1420	6.0	1.00 (referent)	638	3.5	1.00 (referent)
Quartile 3	1126	8.0	0.80 (0.54–1.18)	1002	4.8	1.35 (0.66–2.75)
Quartile 2	862	17.4	1.30 (0.85–1.99)	1324	7.7	1.66 (0.90–3.06)
Quartile 1	532	20.8	1.53 (0.95–2.48)	1918	8.7	1.67 (0.93–3.00)
*P*-trend	3940		0.07	4882		0.09
Education						
Men (*n* = 7894)						
>high school	1352	6.7	1.00 (referent)	1008	5.3	1.00 (referent)
high school	1000	10.5	1.26 (0.97–1.65)	1187	6.9	1.30 (0.76–2.23)
<high school	1073	21.6	1.00 (0.74–1.36)	2274	13.6	1.73 (1.05–2.83)
*P*-trend	3425		1.0	4469		0.02
Women (*n* = 8822)						
>high school	1394	5.6	1.00 (referent)	1116	2.2	1.00 (referent)
high school	1441	9.0	0.95 (0.67–1.33)	1512	4.8	1.64 (0.99–2.72)
<high school	1105	22.6	0.97 (0.70–1.35)	2254	12.0	1.92 (1.14–3.23)
*P*-trend	3940		0.9	4882		0.02

Abbreviations: CI, confidence interval; OR, odds ratio.

^a^Adjusted for age (years), smoking status (never, former, current), current drinker (absent, present), hypertension (absent, present), diabetes (absent, present), cancer (absent, present), cardiovascular disease (absent, present), mean arterial blood pressure (mm Hg), high-density lipoprotein cholesterol (mg/dL), body mass index (kg/m^2^), physical activity (absent, present), and self-rated health (excellent/very good, good, fair/poor).

P-interaction (poverty-income ratio quartiles × sex) was 0.1; P-interaction (education categories × sex) was 0.8.

In a supplementary analysis, we repeated the multivariable models in Table 3 using insurance status as an alternate indicator of SES. The pattern of association was similar to that of PIR. As compared to those with insurance coverage, the multivariable OR (95% CI) of mortality for those without insurance coverage was 1.46 (1.21–1.75), using multivariable model 2.

## DISCUSSION

In a nationally representative sample of US adults, lower PIR—a measure of low SES—was associated with mortality, independent of SRH or demographic, lifestyle, or clinical risk factors. This association between lower PIR and mortality was significant in all racial-ethnic groups. In contrast, an initial association between lower education and mortality that was present after adjustment for demographic factors was not statistically significant after adjusting for lifestyle and clinical risk factors in non-Hispanic whites; however, it remained significant in other race-ethnicities even after multivariate adjustment.

With regards to the income-mortality association, our results are consistent with past studies that identified an independent dose-response relationship between lower income and all-cause mortality.^7^^,^^28^^–^^33^ Our study contributes to the existing literature by showing that income is associated with mortality even after adjusting for SRH and lifestyle and clinical factors, suggesting a relatively independent role. This is similar to the findings from the EPIC-Norfolk study, which showed that occupation has a modest residual effect on mortality even after adjusting for SRH.^19^

Concerning the education-mortality association, past research has mostly been inconclusive regarding the association between lower education and mortality, with more studies reporting an independent association^34^^–^^37^ than not.^7^ Our result that adjusting for SRH and lifestyle and clinical risk factors in the multivariable model mostly explained the education-mortality association in non-Hispanic whites, but not in other racial-ethnic groups, is a new addition to the literature.

In contrast to the education-mortality association, which we found to be mostly explained by SRH and lifestyle and clinical risk factors, the residual association with mortality for income indicates the influence of factors that may not easily be conceived by individuals responding to the question on SRH. These may include contextual factors^38^ or political, economic, cultural, or historic factors^39^ that may shape an individual’s exposure and access to public infrastructure and health care, which are not captured in SRH. Therefore, there is a need for more research in this field to elucidate the role of income on mortality that is not explained by lifestyle and clinical risk factors or SRH.

In non-Hispanic whites, the education-mortality association became statistically insignificant after adjusting for SRH and lifestyle and clinical risk factors. In contrast, among other race-ethnicities, an educational gradient was still apparent after adjusting for SRH and clinical and lifestyle factors. It is possible that increased awareness of the role of lifestyle and clinical risk factors in health among non-Hispanic whites resulted in the narrowing of educational differences in mortality in this population.^40^ In minority ethnicities, it may be that education’s role in health is greater than that perceived and influenced by the individual. In this sense, the residual effects of education in minority ethnicities after adjustment for SRH may be an indicator of differential availability of educational resources across ethnicities in the society. A corollary observation is that health inequalities, including the education-mortality differences that we observed, are still heavily influenced by racial/ethnic differences in the United States. Therefore, it is vital to formulate active federal and state policies that expand health and educational services and opportunities across all race-ethnicities, along with improving attitudes and promoting lifestyle changes.

The association between lower PIR and mortality was significant in both non-Hispanic white men and those of other race-ethnic groups, but not in women. It is possible that men with low income may be exposed to more work-related deaths or indulge in unhealthy lifestyle, including excessive alcohol,^40^ or cigarette smoking.^41^ Our results are consistent with a previous systematic review of observational cohort studies from developed countries, which found that socioeconomic inequalities in mortality were more pronounced in men than in women, using absolute measures of inequality.^42^ However, it is also understood that sex differences in the SES-health association may depend on the SES measure employed in a study and its characteristics (eg, relative or absolute SES terms) and that the association is also highly context-oriented, largely due to differences in cultural or societal characteristics among countries and populations (eg, by age groups).

We found that lack of health insurance coverage—an indicator of access to care—was associated with mortality, independent of SRH or demographic, clinical, or lifestyle risk factors. Two thirds of uninsured persons in the United States are from low-income households that are unable to afford either healthcare insurance or out-of-pocket healthcare costs.^43^ Studies have shown that lack of health insurance is associated with increased risk of mortality due to difficulty in accessing healthcare services and poor quality of healthcare in both preventive services and management of chronic diseases.^44^^,^^45^

An important contribution of our study is the fact that we had adequate sample size and ethnicity-specific data, which permitted detailed stratified analyses by race-ethnicity. We found that even after adjusting for SRH and demographic, lifestyle, and clinical risk factors, PIR was significantly associated with mortality in non-Hispanic whites as well as other race-ethnicities. In contrast, education was independently associated with mortality in other race-ethnicities only. In non-Hispanic whites, the association between education and mortality that was initially evident after adjusting for age and sex was substantially attenuated and lost statistical significance after additional adjustment for lifestyle and clinical risk factors. After additional adjustment for SRH, the magnitude of the association was further weakened. Our results are thus consistent with the hypothesis that various SES variables may interact differently with race-ethnicity in complex ways to cause health effects and higher mortality. For example, our results suggest that lower education is independently related to mortality in racial-ethnic groups other than non-Hispanic whites; whereas, low PIR was related to mortality in both non-Hispanic whites and other race-ethnicities. A corollary observation is that as a public health intervention strategy, improving education may be more beneficial in other race-ethnicities than in non-Hispanic whites, whereas improving income may be equally beneficial in both groups.

There are several limitations in our study. Firstly, lower socioeconomic status has been significantly associated with higher morbidity for chronic diseases such as diabetes,^46^ hypertension,^6^ cardiovascular diseases,^47^ and end-stage renal diseases,^48^ all of which can increase mortality. Our adjustment of these variables may have been inadequate, and residual confounding from these factors may lead to overestimation of the effect of socioeconomic variables on mortality. Secondly, ascertainment of SES, clinical conditions, health behavioral aspects, and SRH was performed only once, at the time of NHANES III. Due to variability in these factors over time, our results may be biased due to time-varying confounding by these factors. Finally, we excluded occupation as measure of SES in our analysis, as it was imprecise among women and retirees in the sample. However, we believe that exclusion of occupation status from the analyses is unlikely to have biased the study findings, as it was previously established that income might be a better SES indicator of health, given that employment status is a subset of income.^20^ The availability of a large and nationally representative sample, along with rich covariate information, is a major strength of our study.

In conclusion, we found that lower PIR was associated with mortality in all racial-ethnic groups, independent of SRH or demographic, lifestyle, or clinical risk factors. In contrast, lower education was not significantly associated with mortality in non-Hispanic whites, although the association remained significant in other race-ethnicities. While our results suggest that adjusting for SRH resulted in only a modest change in the SES-mortality association beyond lifestyle and clinical risk factors, our findings also highlight the significance of racial-ethnic variations in socioeconomic disparities in health in relation to education.
